# Topical antimicrobial agents for pediatric burns

**DOI:** 10.1186/s41038-017-0096-6

**Published:** 2017-10-19

**Authors:** Robert Cartotto

**Affiliations:** Ross Tilley Burn Centre, Sunnybrook Health Sciences Centre, University of Toronto, Rm D 712, 2075 Bayview Avenue, Toronto, ON M4N 3M5 Canada

**Keywords:** Burns, Topical antimicrobial, Infection, Dressings, Wound, Pediatric

## Abstract

While topical antimicrobial agents are indicated for most if not all burn wounds, the choice of a topical agent must consider many factors such as the wound depth, anticipated time to healing, need for surgical intervention, and the known cytotoxicity of the agent. Especially relevant to the pediatric burn patient are the antimicrobial agent’s properties related to causing pain or irritation and the required frequency of application and dressings. This article will discuss the general principles surrounding the use of topical antimicrobials on burn wounds and will review the most common agents currently in use.

## Background

Topical antimicrobial agents for the burn wound were developed in the 1950s and 1960s to deal with the problem of invasive infection of the burn wound. In that era, deeper burn wounds were treated by gradual debridement of the burn eschar using immersion hydrotherapy, and topical antimicrobial agents were integral to this approach to help control microbial proliferation in the wound. Invasive infection of the burn wound leading to sepsis and death was commonplace [1]. Aside from the recognized threat of burn wound sepsis, burn wound infections also may lead to wound conversion, skin graft failure, and prolonged hospitalization. The introduction of topical antimicrobial agents was a major advancement in burn care and proved to be responsible for important reductions in mortality from burn wound sepsis [2, 3]. Currently, while the problem of invasive burn wound infection has largely been eliminated by early surgical excision and closure of deep second-degree and third-degree burns, topical antimicrobial control in these wounds prior to definitive surgical debridement is still necessary. Even superficial burns which are expected to heal may benefit from the use of topical antimicrobial agents since microbial proliferation in a burn wound has the potential to significantly delay healing [4], the main consequence of which is increased scarring. Therefore, regardless of burn depth, topical antimicrobials are most importantly indicated when there is clinical suspicion of risk of infection, or when a wound infection is evident.

Paradoxically, many of the topical antimicrobial agents currently in use also have cytotoxic effects on keratinocytes and fibroblasts and have the potential to delay wound healing. Thus, while topical antimicrobial agents are indicated for most if not all burn wounds, the choice of a topical agent must consider many factors such as the wound depth, anticipated time to healing, need for surgical intervention, and the known cytotoxicity of the agent. Especially relevant to the pediatric burn patient are the antimicrobial agent’s properties related to causing pain or irritation and the required frequency of application and dressings. This article will discuss the general principles surrounding the use of topical antimicrobials on burn wounds and will review the most common agents currently in use.

## Review

### General principles

#### Microbiology of the burn wound

The appearance of microbes in the burn wound follows a predictable pattern. Initially, but only transiently, the wound is sterile. Within 48 h of injury, gram-positive bacteria that are normally found in the skin such as *Staphylococcus aureus*, *Corynebacterium*, and *Streptococcus* species colonize the wound surface. By 5–7 days post-burn, other organisms originating from the patient’s normal gastrointestinal or respiratory flora, or the hospital environment, appear and begin to dominate. These are usually gram-negative organisms such as *Pseudomonas aeruginosa*, *Enterobacter* species, *Proteus*, and *Escherichia coli*. Unfortunately, the full spectrum of emerging antibiotic-resistant bacteria including methicillin-resistant *Staphylococcus aureus* (MRSA) and multiresistant *Acinetobacter* are now frequently encountered in the burn wound at this stage. Later, yeasts and fungi may appear [5, 6], which is always an ominous sign connected with heightened mortality [7].

#### Colonization and infection

While almost all burn wounds will become colonized with microorganisms, this does not always cause harm. Colonization should be distinguished from a burn wound infection, in which large numbers of bacteria (> 105 organisms/gm of tissue) populate the wound and produce clinically apparent disease that features local signs and symptoms (e.g., surrounding redness, pain, swelling, wound discoloration, and early eschar separation) as well as systemic manifestations (e.g., fever, leukocytosis, sepsis) [8–10].

Since superficial burns have a preserved blood supply and perfusion through much of the dermis, they typically will become colonized but less frequently develop invasive burn wound infections. In contrast, deeper burns are covered by an avascular layer of moist and protein-rich dead skin (the eschar), which fosters bacterial proliferation and invasion, leading to burn wound infection. Furthermore, generalized immunosuppression associated with major burn injuries predisposes the patient to local burn wound infection. When bacteria in the eschar penetrate surrounding uninjured tissues and invade the bloodstream, fatal sepsis may result. Hence, there is an important need to suppress bacterial growth with topical agents, especially in deeper burns, to prevent invasive burn wound infection and its life-threatening consequences.

#### Infection impairs burn wound healing

Infection will delay wound healing [11]. Bacteria produce numerous endotoxins, exotoxins, and proteases which inflict further tissue injury. The microbial population also has metabolic requirements and consumes resources necessary for wound healing [4]. Finally, a heavy bioburden stimulates an increased inflammatory response, the by-products of which may cause injury to healthy tissue [4]. These problems are particularly germane to more superficial burns that are attempting to heal spontaneously and provide the rationale for the use of topical antimicrobial agents in this setting.

#### Topical antimicrobials also may impair wound healing

Many topical antimicrobial agents are cytotoxic to keratinocytes and fibroblasts, and as such have the potential to delay wound healing [12, 13]. It is not surprising that in systematic reviews of controlled trials comparing biosynthetic skin substitutes to topical antimicrobial dressings for superficial partial-thickness burns, faster healing was observed with the use of skin substitutes [14, 15]. Consequently, the choice of a topical antimicrobial agent must be a delicate balance between the need to control microbial growth in the burn wound, and the potential risk that the topical agent may impair wound healing. In practical terms, among more superficial burns that are expected to heal on their own, it is more important (and difficult) to strike this balance. In these burns the goal is healing within 2–3 weeks of injury to reduce the likelihood of hypertrophic scarring [16]. Conversely, in a deeper burn that is not expected to heal spontaneously and which will be excised and closed surgically, there is a greater emphasis on suppressing microbial growth and less emphasis on optimizing conditions for spontaneous healing.

#### Topical trumps systemic delivery

Systemic antimicrobial drugs are not recommended because they are ineffective against colonization and infection of the burn wound [15]. Avascular eschar and the presence of biofilms are the main impediments that limit the delivery and effectiveness of systemic antimicrobials, and the routine use of systemic agents only leads to the emergence of dangerous multiresistant microbial strains. In contrast, topical antimicrobials are delivered directly to the burn wound, and to varying degrees penetrate eschar and limit the development of infection. Although microorganisms are capable of developing resistance to topical agents, this is much less common than to systemic antibiotics. This may be in part related to the route of delivery. However, one study found that while many multidrug-resistant organisms (MDROs) are susceptible to commonly used topical agents, higher rates of resistance were seen than to non-MDROs [17]. While antimicrobial resistance to topical antimicrobials is less common than to systemic agents, practitioners should always consider this possibility as well as strategies to deal with this problem. One approach is to know the common or endemic organisms within the burn care facility and to avoid use of topical agents that are ineffective against those microbes. For example, where fungus is endemic, mafenide acetate may not be a good choice due to its inactivity against fungus. Another strategy may be to rotate use of various topical agents rather than employ only one agent.

#### The ideal topical antimicrobial does not exist

The ideal topical antimicrobial for burn wounds would have the following properties: It would have a broad spectrum of coverage and would not stimulate the development of resistance. It would be able to penetrate well into the burn eschar while being painless to apply and requiring infrequent dressing changes or reapplication. Finally, it would not inhibit wound healing and would be non-absorbable and free of systemic adverse effects. Unfortunately, none of the currently available topical antimicrobial agents meet all these criteria.

### The common topical antimicrobial agents

#### Silver-based agents

##### Silver physiology

Silver has been known for centuries to have antimicrobial properties, and it is the foundation of established topical antibacterial agents for the burn wound such as silver nitrate solution, silver sulfadiazine cream, and silver-releasing dressings. Metallic silver (Ag^0^) is biologically inert and has no antimicrobial activity, but the silver cation (Ag^+^) is highly reactive and strongly bactericidal, at relatively low concentrations. Silver may also exist in two highly reactive and unstable oxidation states: Ag^++^ and Ag^+++^ [18]. Silver ions are toxic to bacteria, yeasts, and fungi through several mechanisms. These include inhibition of enzymes necessary for metabolism and respiration of the microorganism, disruption of the cell membrane or cell wall of the microbe, and interference with DNA and RNA preventing replication of the microorganism [18–20]. Microbial killing is strongly correlated with the concentration of free silver ions [18]. However, free Ag^+^ is rapidly bound and depleted by proteins and compounds on the wound surface and in the wound fluid. This hinders the maintenance of adequate Ag^+^ levels necessary for microbe killing on the wound bed. Resistance to silver is uncommon, presumably because silver acts by multiple mechanisms, but there is some evidence that suggests that chronic exposure to very low concentrations of ionic silver can induce resistance. Thus, it is recommended that dressings or agents that release high levels of ionic silver are preferable, from the standpoint of avoiding development of silver resistance [4].

Although ionic silver is an effective antimicrobial agent, in vitro studies have found that it is also cytotoxic to cells essential for wound healing such as keratinocytes and fibroblasts, and silver has been shown to delay healing of second-degree burns in vivo [20–25]. Therefore, silver’s potential to slow re-epithelialization should always be considered before using a silver-based agent on partial-thickness burns that are expected to heal spontaneously.

##### Silver nitrate

A 0.5% silver nitrate (AgNO_3_) solution has been used as a topical antimicrobial agent for burn wounds for over half a century [26]. Ionic silver dissociates from AgNO_3_ to effectively inhibit a broad spectrum of microorganisms on the burn wound including *Staphylococcus* species, some gram-negatives including *Pseudomonas* and some yeasts. However, the liberated free silver ions readily precipitate with chloride and any other negatively charged molecules, inactivating the silver, and creating inert silver salts. Consequently, silver ions do not penetrate deeply into the eschar and must be frequently replenished by keeping the gauze dressings on the wound continuously wet with the 0.5% AgNO_3_ solution. Furthermore, these silver salts stain everything that they contact, from the wounds to the dressings to the patients’ bed linens and room surfaces, with a brown-black residue. Poor eschar penetration and labor intensiveness are considered the main drawbacks of AgNO_3_. Also, the margin between silver nitrate’s antimicrobial activity and cytotoxicity is narrow; Moyer recognized that a 1% concentration of AgNO_3_ harmed re-epithelialization of partial-thickness burns [26]. Furthermore, as the silver precipitates the remaining free water constantly in contact with the wound, it can cause hyponatremia and hypochloremia when AgNO_3_ is applied to large surface areas, so it is important to monitor the patient’s electrolytes when this material is used. Bacterial conversion of nitrate to nitrite may rarely lead to methemoglobinemia [26].

##### Silver sulfadiazine

Silver sulfadiazine (SSD) is applied universally as a topical antimicrobial for burns. It is a water-soluble cream containing 1% silver sulfadiazine. This agent’s main effect comes from the continuous dissociation and deposition of silver ions on the wound surface; the sulphadiazine component, while having a bacteriostatic effect, plays a secondary role. Silver sulfadiazine is effective against numerous microorganisms commonly found in the burn wound including gram-positive bacteria (e.g., *Staphylococcus aureus*, *Streptococcus pyogenes*, *Corynebacterium diptheriae*, *Clostridium perfringens*), gram-negative bacteria (e.g., *Pseudomonas aeruginosa*, *Klebsiella* species, *Enterobacter* species, *Proteus* species, *Citrobacter*, and *Escherichia coli*), as well as *Candida albicans* and other fungi [27, 28].

One of the main drawbacks of SSD is its potential to impair epithelialization and wound healing due to the silver’s cytotoxic effect on fibroblasts and keratinocytes. This effect has been observed in many clinical studies where SSD was compared to alternative dressings or topical antimicrobials [14, 29]. While much of this evidence is low quality, there seems to be a consistent pattern showing that SSD delays healing of superficial burns [14]. The other important drawback of SSD is that it forms an amalgamate with surface proteins from the wound to create a pasty yellowish-white exudate on the wound surface, referred to as a “psuedoeschar”, which obscures visualization of the wound surface and which may be mistaken for the true eschar of a deeper burn.

A minority of patients experience cutaneous hypersensitivity to SSD, and the agent cannot be used in patients who are allergic to sulfonamides. Application to the burned face is relatively contraindicated due to the risk of ocular irritation or injury. Because of the risk of kernicterus from the sulfonamide component, SSD should not be used in infants < 2 months of age or during pregnancy. While silver is readily absorbed, systemic silver toxicity to specific organs such as the liver or the kidney through silver deposition is exceedingly rare but theoretically should be considered when SSD is repetitively applied to large surface areas [30]. Finally, SSD has a relatively short duration of action and penetrates only the superficial part of the burn eschar [31]. Therefore, SSD may need to be reapplied more than once per day to preserve a sufficient reservoir of the compound to maintain continuous dissociation of silver onto the wound surface, although daily vs more than once-daily application of SSD has never been formally studied. This has implications for all burn patients but especially children who would be subjected to repetitive painful dressing changes when this agent is chosen.

##### Silver-releasing dressings

The latest way to deliver silver to the burn wound is the silver-releasing dressing. There are numerous silver-releasing dressings which can be broadly classified as follows [19, 32, 33]:

- Nanocrystalline dressings are densely coated with nanocrystals (< 20 nm in diameter) each containing 30–50 silver atoms. When moistened, the dressing produces a sustained release of Ag^+^ and uncharged Ag^0^.
- Hydrocolloid and Hydrofiber silver dressings have silver bound to the hydrocolloid or carboxymethylcellulose Hydrofiber, respectively, and produce a gradual sustained release of Ag^+^ as the dressing absorbs fluid.
- Activated charcoal dressings with silver work by adsorbing bacteria into the dressing where they are then destroyed by silver in the dressing.
- Silver foam dressings.

In vitro, nanocrystalline silver dressings have shown antimicrobial activity against a broad spectrum of bacteria, antibiotic-resistant organisms, as well as yeasts and fungi [34–36]. A major advantage of these dressings is that their sustained release of ionic silver provides an effective antimicrobial effect while potentially requiring fewer painful dressing changes as compared to more traditional approaches such as silver nitrate dressings [37]. This might be especially beneficial in the pediatric burn population. Silver-releasing dressings such as Aquacel® Ag, a hydrocolloid silver dressing, can be left intact on partial-thickness burns for up to 2 weeks, significantly reducing the number of dressings, painful wound manipulations, nursing time, and hospital length of stay in children with partial-thickness burns [38, 39]. Similar findings of reduced hospitalization and cost by use of outpatient nanocrystalline silver dressings as opposed to inpatient SSD for pediatric patients with scald burns have been reported [40]. At present, there is insufficient evidence from randomized clinical studies (which predominantly involve partial-thickness burns) to confidently determine that silver-releasing dressings prevent burn wound infections [41]. Similarly, there is conflicting evidence on whether silver-releasing dressings impede or promote re-epithelialization [42–44].

#### Mafenide acetate

Mafenide acetate (Sulfamylon®, Mylan Inc. Canonsburg PA, USA) is a topical sulfonamide antibiotic that can penetrate deep into eschar and tissues, and it is active against many gram-positive and gram-negative organisms. This capability was originally harnessed to successfully counter the problem of invasive burn wound infection and fatal septicemia from gram-negative species, especially *Pseudomonas* [2, 3]. The agent was initially produced as an 11% cream, but is also available as a 5% aqueous solution. The most common use of mafenide acetate (MA) is for deep or infected burns where penetration of the antibiotic into the eschar is advantageous. For the same reason, the cream is also used for deep burns of the ear to prevent invasive infection leading to suppurative chondritis of the ear cartilage [45]. More recently, 5% and even 2.5% MA solution have been used in all phases of burn wound care including application to unexcised burns and as a postoperative irrigation on freshly applied skin grafts [46, 47].

One problem with MA is its lack of antifungal activity. Addition of nystatin to MA is used to avoid fungal overgrowth with prolonged use of MA. Another disadvantage is that MA is painful on application, especially on more superficial wounds. To some extent, this problem has been reduced by using the 5 and 2.5% solutions [46, 47]. Like other topical antimicrobials, MA is cytotoxic to fibroblasts and keratinocytes and may impede wound healing. In vitro studies suggest that concentrations as low as 0.1% are toxic to these cells [23]. Another adverse effect is that MA is a carbonic anhydrase inhibitor and may cause severe metabolic acidemia with compensatory hyperventilation when it is repetitively applied to large surface areas. For this reason, mafenide acetate cream is usually reserved for smaller deep burns, or it is alternated with SSD on larger burns. Acid-base disturbances were not seen with use of the 5% solution in a study of nearly 700 adult and pediatric burn patients [46]. Finally, MA may occasionally cause a local rash or skin irritation [48, 49].

#### Antibiotic ointments

An antibiotic ointment contains an antibiotic within a water-in-oil emulsion where the volume of oil exceeds that of the water. Thus, such ointments provide not only an antibacterial effect but also they create a moist wound healing environment. Hence, these agents are optimally suited for superficial burns where spontaneous healing is expected. While the spectrum of bacterial coverage tends to be limited, these agents are relatively free of complications. In general, the ointments are applied two to three times daily as a thick layer for moisture retention and then are covered with a non-adherent dressing layer followed by gauze [48]. Mostly they are soothing to apply, easier to clean off than creams such as SSD, and tend to be reasonably well tolerated by children.

##### Bacitracin

Bacitracin is a topical agent effective against gram-positive bacteria but not gram-negative bacteria or yeasts. Bacitracin ointment is contained in a petroleum base which helps to maintain a moist wound healing environment. Usually, bacitracin is applied to superficial burns, especially those on the face. Due to the lack of fungal coverage, prolonged use, especially after re-epithelialization has occurred, may lead to overgrowth of yeast causing a rash. Bacitracin should therefore be terminated as soon as the wound has epithelialized [48, 49].

##### Polymixin B sulfate

Like Bacitracin, Polymixin B sulfate is impregnated in a thick petroleum-based ointment that helps with moisture retention. The antibacterial spectrum covers many gram-negative bacilli including *Pseudomonas*, but activity against gram-positives is limited. Absorption and systemic toxicity such as nephrotoxicity or neurotoxicity are uncommon but could be seen with repeated application to large surface areas [5].

##### Neomycin

This aminoglycoside antibiotic ointment covers gram-negative bacilli such as *Escherichia coli* and *Enterobacter*, along with some gram-positive species. Unlike the other antibiotic ointments, bacteria tend to develop resistance to neomycin more frequently and local skin irritation is seen more frequently. Absorption following application to large surface areas may lead to systemic toxicity including nephrotoxicity and ototoxicity [48, 49].

##### Combination ointments

The limited antibacterial spectrum of the individual agents described above is partly overcome by combining them. Typical examples are Polysporin® (Johnson and Johnson, New Jersey, USA) which combines bacitracin and polymixin B sulfate, and Neosporin® (Johnson and Johnson, NJ, USA) which combines bacitracin, polymixin B sulfate, and neomycin.

##### Mupirocin

This topical agent is highly effective against gram-positive skin flora including *Staphylococcus aureus*, and importantly, it is the only topical ointment that can suppress MRSA. The frequent emergence of MRSA in burn units has led to widespread use of this agent for MRSA-colonized or infected burn wounds [5].

#### Antiseptic solutions

Antiseptic solutions are chemical agents that are externally applied to wounds and tissues. These agents typically have a broad spectrum of activity and act through multiple simultaneous mechanisms, which may be the reason that microorganisms do not develop resistance to these agents as readily as to antibiotics. Many antiseptic solutions are also able to disrupt biofilms [50]. Thus, these agents were originally used on chronic wounds but more recently they have been utilized for microbial control on acute burn wounds. Most of these agents are cytotoxic to keratinocytes and fibroblasts and can impair wound healing. In general, the optimal solution concentration that provides an acceptable balance between microbial killing and avoidance of cytotoxicity is unknown for most of these agents.

##### Hypochlorous solutions

Sodium hypochlorite (NaOCl) solutions are mainly represented by Dakin’s solution which is buffered 0.5% NaOCl. Dakin’s solution is widely effective against most bacteria including multidrug-resistant organisms, fungi, and viruses. Concentrations as low as 0.025 to 0.00025% have been found in vitro to be effective [12, 51]. However, in vitro cytotoxicity to fibroblasts and keratinocytes has also been reported in this concentration range [12, 13, 51]. Heggers et al. have stated that 0.025% Dakin’s solution is an optimal concentration that was effective against all tested bacterial strains and which did not produce significant cytotoxicity [51]. Use of unbuffered sodium hydroxide (NaOH) at 0.006% has been reported to be effective in vitro and not toxic to fibroblasts [52]. Since the action of NaOCl is short lived, the method of Carrel was used originally to continually drip the solution into the wound dressings. This approach seems to have been abandoned and the solution is now applied two to three times a day as gauze-soaked dressings. Because of the potential for cytotoxicity, this agent would mostly be applied to deep burns that are not expected to heal prior to surgical excision, or to chronic wounds especially if a biofilm is present.

##### Acetic acid

Acetic acid solution appears to have activity against common burn wound pathogens including those contained within biofilms [53]. Once again, the appropriate concentration that optimizes bacterial eradication and minimizes cytotoxicity to keratinocytes and fibroblasts is unknown. Concentrations of 0.25% are cytotoxic to cultured keratinocytes in vitro [54] while acetic acid solutions in clinical use generally range between 1 and 3%. Given that this agent is cytotoxic, one might consider reserving this agent for deeper burns that are not expected to heal spontaneously and which are anticipated to require surgical excision, or on chronic infected wounds, rather than more superficial wounds where one expects spontaneous healing by re-epithelialization.

##### Chlorhexidine

Experience with 0.05% chlorhexidine gluconate for burn wounds is limited [55] and use of 0.5% chlorhexidine diphosphanilate cream was found to be difficult and painful to apply on burn wounds [56]. The addition of 0.2% chlorhexidine to SSD was found to be especially cytotoxic to keratinocytes in vitro [24] and significantly delayed healing of second-degree burns when compared to paraffin gauze alone [57]. There is little to support the use of this agent in the pediatric burn population.

#### Cerium nitrate

Although early debridement and closure are strongly recommended for deep dermal and full-thickness burns, there are situations where early surgical excision cannot be performed. Under these circumstances, the application of cerium nitrate (CN), a salt compound of the rare earth element cerium, to these wounds may be beneficial. The application of CN has two effects. The first is that application turns burn eschar into a dry, hard, and adherent “shell” that protects the underlying wound from bacterial invasion. Eventually, when surgical excision of this cerium-hardened eschar is performed, the underlying granulation tissue is typically clean and suitable for grafting upon. The second effect is that cerium binds and inactivates the release of lipid protein complex which is a pro-inflammatory and immunosuppressive toxin produced when heat polymerizes skin proteins [58]. Originally, patients were bathed in a solution of CN or had gauzes soaked in CN applied to their wounds, but nowadays, CN is usually applied as a cream that combines 2.2% CN with 1% silver sulfadiazine (Flammacerium® Solvay SA, Brussels, Belgium). A recent uncontrolled retrospective study involving over 800 patients with a mean ± SD percent total body surface area (%TBSA) burn of 6.7 ± 11.2 reported that cerium nitrate-SSD application allowed safe deferral of surgical burn wound excision especially in children and the elderly [59]. However, older literature has found conflicting results with respect to CN’s effects on mortality [60–62].

#### A practical approach

All burn wounds in children are initially treated by cleansing of the wound followed by application of a topical antimicrobial agent. The choice of an agent is complicated by the wide variety of products that are available. The decision must consider the depth and age of the burn, whether there are clinical signs of infection, the location of the burn, and most importantly whether the burn is expected to heal spontaneously or whether surgical excision is anticipated. In all cases, the goal is to achieve a stable healed wound within 2–3 weeks of injury.

##### First-degree burns

These burns are not at risk of infection and do not require topical antimicrobial agents. They should be kept clean and moisturized.

##### Second-degree (partial-thickness) burns

Superficial partial-thickness burns are expected to heal within 2 weeks, and the goal here is to optimize conditions for rapid epithelialization. These conditions are, first, to maintain a moist environment and second, to avoid cytotoxicity to keratinocytes. Hence, most of the standard topical antimicrobials such as SSD, silver nitrate, mafenide acetate, and the antiseptic solutions are not ideal. These agents are effective antimicrobials but all appear to have the potential to inhibit wound healing. The risk to benefit ratio with these agents for a superficial dermal burn is high.

A preferable approach, after cleansing of the wound, is the application of an antibacterial ointment such as bacitracin, neomycin, or a combination agent. After applying a thick layer of one of these ointments, the wound is covered with a non-adherent dressing (e.g., paraffin gauze, Xeroform®, or Adaptic®) followed by bulky gauze. The main disadvantage of this approach is that two or three times a day the dressing needs to be removed and the wounds must be cleansed and old ointment removed before applying a new dressing. This is usually painful and traumatic for the child and utilizes resources. An alternative approach is to consider one of the nanocrystalline silver-releasing dressings, which can be left in place for much longer periods thus reducing (or eliminating) routine dressing changes. While silver is considered cytotoxic to keratinocytes, there is insufficient evidence at present to prove that the nanocrystalline silver-releasing dressings inhibit healing of second-degree burns.

The deep second-degree burn in a child poses a more difficult challenge. The difficulty mainly arises from our imprecision in diagnosing this burn depth. If the burn is actually not as deep into the dermis as clinically suspected, there is a possibility of spontaneous healing within the 2 to 3 week time limit, but this could be potentially impaired with use of some of the common topical antimicrobial agents such as SSD or mafenide acetate. However, If the burn is truly a deep partial-thickness wound, there is a higher risk of a burn wound infection and early excision and grafting is the recommended approach. In this case, there is less concern over inhibiting spontaneous healing, and the risk to benefit ratio of standard topical antimicrobials such as silver nitrate, SSD, and mafenide acetate is lower. One practical consideration in this scenario is that SSD and mafenide cream leave a pseudoeschar on the wound which makes ongoing assessment of the burn depth even more difficult. This problem could be avoided with the 5% mafenide acetate solution. Antiseptic solutions such as Dakin’s or acetic acid may also be considered but are less conventional. Nanocrystalline silver-releasing dressings such as Acticoat® may also be a useful option as they require less frequent changes and do not produce a pseudoeschar.

##### Third-degree (full-thickness) burns

Third-degree burns ideally will undergo early surgical excision and closure. Here, the goal is to provide effective antimicrobial control to prevent invasive infection of the burn wound before surgical excision. Antimicrobial creams such as SSD or mafenide acetate are usually applied in this situation. These agents require daily or twice-daily removal, reapplication, and re-dressing, which will necessitate appropriate analgesia, sedation, and the associated resources to provide this safely to a child. Nanocrystalline silver dressings are an alternative and have the advantage of reducing the number of dressing changes since these materials can be left intact for several days if they are kept moist.

## Conclusions

Burn wound infection has many undesirable consequences including delayed healing leading to worsened scar formation, invasive infection leading to sepsis and death, prolonged hospitalization, and increased economic costs. Application of a topical antimicrobial agent to a burn wound is now a standard intervention that contributes to improved outcome following burn injury. However, the wide variety of available agents makes the choice of an appropriate agent quite challenging, especially in children with burns. Ultimately, a delicate balance must be struck between the need to control microbial proliferation in the burn wound, and the avoidance of impaired wound healing that can be caused by many of the available agents, while simultaneously giving careful attention to the ease and frequency of application of the agent. In general, antimicrobial ointments such as bacitracin, polymixin B sulfate, or a combination ointment, or hydrocolloid and hydofiber nanocrystalline silver dressings appear to be most suited to superficial second-degree burns. Topical agents such as silver sulfadiazine cream, mafenide acetate cream, nanocrystalline silver dressings, and hypochlorous antiseptic solutions are recommended for deep second- and third-degree burns prior to early surgical excision and closure.

## Abbreviations

Ag^+^, Ag^++^, Ag^+++^: Oxidation states of silver (ionic silver)
Ag^0^: Inert metallic silver
AgNO_3_: Silver nitrate
CN: Cerium nitrate
MA: Mafenide acetate
MDRO: Multidrug-resistant organism
MRSA: Methicillin-resistant *Staphylococcus aureus*
NaOCl: Sodium hypochlorite
NaOH: Sodium hydroxide
SSD: Silver sulfadiazine
%TBSA: Percent total body surface area
